# Blink Behaviour, Pupil Diameter, and Technical Performance in Elite Male Table Tennis Players: A Wearable Eye-Tracking Study

**DOI:** 10.3390/sports14100420

**Published:** 2026-10-01

**Authors:** Aitor Centeno, Rut González-Jiménez, Ricardo Bernárdez-Vilaboa, Juan E. Cedrún-Sánchez, Miaoyu Han, Francisco Javier Povedano-Montero

**Affiliations:** 1 GICAF Research Group, Research Methods and Evaluation Department, Faculty of Human and Social Sciences, Universidad Pontificia Comillas, 28015 Madrid, Spain; 2 Optometry and Vision Department, Faculty of Optics and Optometry, Complutense University of Madrid, 28037 Madrid, Spain; 3 Applied Vision Research Group, Faculty of Optics and Optometry, Universidad Complutense de Madrid, 28037 Madrid, Spain; 4 Faculty of Physical Activity and Sport Sciences, Universidad Politécnica de Madrid, 28040 Madrid, Spain; 5 Hospital Doce de Octubre Research Institute (i+12), 28041 Madrid, Spain

**Keywords:** table tennis, wearable eye tracking, blink frequency, pupil diameter, visuomotor performance, stroke accuracy, perceptual-cognitive regulation

## Abstract

Background: Table tennis is a fast interceptive sport that requires continuous visual information processing and precise visuomotor control. Wearable eye tracking may provide objective information about ocular behaviour during sport-specific performance, although evidence on blink behaviour and pupil diameter in elite players remains limited. Methods: This observational cross-sectional study analysed blink-related and pupil-related variables during a standardised table tennis stroke-performance test in 17 elite male players. Participants completed a 56-ball robot-based protocol, consisting of six familiarisation trials followed by 50 analysed forehand topspin strokes. Ocular variables included total blink count, blink frequency, mean blink duration, mean pupil diameter, maximum pupil diameter, and minimum pupil diameter. Technical performance outcomes were stroke accuracy and ball velocity. Results: Blink frequency showed the strongest positive association with stroke accuracy score (Spearman’s ρ = 0.613, 95% CI [0.060, 0.881], *p* = 0.012), although this association did not remain statistically significant after false discovery rate correction (FDR-adjusted *p* = 0.115). Total blink count showed a similar association that did not reach statistical significance (ρ = 0.496, *p* = 0.051). No significant associations were observed between blink-related variables and ball velocity. Pupil-diameter variables were not significantly associated with either stroke accuracy or ball velocity. Conclusions: Blink-related measures showed the clearest relationship with stroke accuracy, whereas global pupil-diameter measures did not show comparable associations with technical performance. These findings should be interpreted as exploratory and hypothesis-generating and require confirmation in larger samples using time-resolved analyses of ocular behaviour.

## 1. Introduction

Table tennis is a fast interceptive sport in which successful performance depends on the continuous integration of perceptual, cognitive, and motor processes. Players must extract relevant visual information from the opponent, the racket–ball contact, and the early ball trajectory, and transform this information into accurate motor responses within very short time windows. Therefore, technical performance in table tennis cannot be explained only by physical or biomechanical factors, but also by the efficiency with which players perceive, select, and use visual information during sport-specific actions [1,2,3].

The perceptual demands of table tennis have increased in parallel with the evolution of the modern game, which is characterised by faster rallies, higher stroke velocities, and greater tactical complexity [4]. In this context, expert players are required to anticipate future events before all relevant information is fully available. Previous research in racket and striking sports has shown that skilled athletes are generally better than less experienced players at using early visual cues, anticipating ball direction, and adapting their motor response to time-constrained situations [1,2,3]. In table tennis specifically, visual strategies involving gaze, eye, and head movements appear to be highly task-dependent and may contribute to effective ball tracking during rallies [5]. Recent evidence also suggests that table tennis players may show enhanced saccadic performance when tracking moving targets, probably as a result of extensive visuomotor experience in the sport [6].

Eye-tracking technology has become an increasingly useful tool for studying perceptual-cognitive expertise in sport. Early research mainly focused on visual search behaviour, fixation patterns, gaze location, and anticipatory cues. More recently, wearable and applied eye-tracking systems have allowed researchers to examine visual behaviour and visuocognitive performance in more representative and applied sport settings. Previous work in rhythmic gymnastics has shown that eye-tracking-derived visual variables can contribute to the assessment and modelling of sport-specific visual performance, supporting the relevance of objective oculomotor metrics in athlete evaluation [7,8]. This is especially relevant in table tennis, where laboratory-based tasks may not fully reproduce the temporal constraints and dynamic visual demands of realistic sport-specific situations. Recent studies using wearable eye tracking have begun to describe visuomotor strategies in table tennis players under naturalistic conditions, suggesting that portable systems can provide valuable information about gaze behaviour and visual processing during sport-specific actions [9].

Although most eye-tracking studies in sport have focused on gaze direction, fixations, or quiet eye duration, other ocular variables may also provide relevant information about the cognitive and attentional state of the athlete. Spontaneous blinking, for example, is not only a physiological mechanism related to ocular surface protection, but has also been associated with attention, fatigue, information processing, and task demands [10,11]. Blink behaviour may vary depending on the need to maintain visual input, the temporal structure of the task, and the cognitive resources allocated to performance. In dynamic sports such as table tennis, where relevant visual information is available only for very brief periods, blink behaviour could be particularly informative.

Pupil diameter is another ocular variable that has been widely used as a physiological marker of cognitive load, mental effort, and attentional engagement [12,13]. Increases in task difficulty or processing demands are frequently accompanied by changes in pupil size, although pupillary responses are also sensitive to illumination, emotional arousal, fatigue, and other contextual factors. For this reason, pupillometry in sport-specific settings requires careful methodological control, especially when wearable eye-tracking systems are used outside strictly controlled laboratory conditions. Nevertheless, pupil-based metrics may provide complementary information about how athletes manage perceptual and cognitive demands during technical performance tasks.

Despite growing interest in eye tracking in sport, evidence on blink behaviour and pupil dynamics during table tennis performance remains limited. Previous studies using wearable eye tracking in table tennis have primarily examined gaze location, eye and head movements, visual search strategies, and differences associated with expertise [5,6,9]. Blink behaviour and pupil diameter may provide complementary information because they reflect aspects of ocular and physiological function that are not captured by gaze location alone. Blinking involves brief interruptions of visual input and is influenced by both physiological and task-related factors, whereas pupil diameter is sensitive to changes in arousal, attentional demand, and cognitive effort. Examining these variables during a standardised sport-specific task may therefore extend current knowledge beyond spatial gaze behaviour and help determine whether additional ocular metrics are related to objective measures of technical performance.

This gap is relevant because variables such as ball velocity and stroke accuracy are commonly used to assess sport-specific performance in table tennis and may be affected by perceptual-cognitive demands, fatigue, or attentional regulation [14,15].

From a methodological perspective, studying elite athletes in sport-specific settings is particularly relevant, although it often involves limited sample sizes due to the restricted accessibility of this population. In this context, ecologically valid protocols may provide complementary information to laboratory-based assessments by capturing ocular responses during tasks that preserve the temporal and technical constraints of the sport. This balance between ecological validity and experimental control has been identified as a central challenge in sport-related eye-tracking research [16]. However, the use of pupil- and blink-related metrics in dynamic settings also requires cautious interpretation, because these variables can be influenced by task structure, lighting conditions, fatigue, signal quality, and individual variability [17]. Therefore, examining these measures during a standardised table tennis test may help to clarify their potential usefulness as complementary indicators of perceptual-cognitive demands and technical performance in elite players.

Therefore, the aim of the present study was to analyse blink behaviour and pupil diameter recorded with wearable eye-tracking technology during a sport-specific table tennis test in elite male players and to explore their associations with objective indicators of technical performance, namely stroke accuracy and ball velocity. We hypothesised that blink-related and pupil-related variables would be associated with technical performance outcomes during the sport-specific task. Given the limited evidence available for these ocular measures in elite table tennis, the analyses were considered exploratory.

The study was not designed to compare ocular behaviour across different levels of sporting expertise, but rather to examine performance-related associations within a highly selected elite population.

## 2. Materials and Methods

### 2.1. Study Design

This observational cross-sectional study was designed to examine associations between ocular variables recorded with wearable eye-tracking technology and sport-specific technical performance within a sample of elite male table tennis players. The study was not designed as a between-group comparison of sporting expertise. Each participant completed a single experimental session in which ocular behaviour and technical performance were recorded simultaneously during a standardised table tennis stroke–performance test.

The ocular variables analysed were total blink count, blink frequency, mean blink duration, mean pupil diameter, maximum pupil diameter, and minimum pupil diameter. Technical performance outcomes included stroke accuracy and ball velocity. All assessments were conducted in the table tennis hall of the High Performance Centre (Centro de Alto Rendimiento, CAR) in Madrid, Spain, under indoor conditions without exposure to natural light. To reduce the influence of external factors on ocular recordings, all participants were evaluated under similar environmental conditions and within the same time period.

### 2.2. Participants

Seventeen male elite table tennis players voluntarily participated in the study. All participants had international competitive experience representing their national teams and regularly competed in the highest national categories, including the Spanish Superdivision and Honor Division, as well as in international competitions. The sample included 14 senior international players, one U19 international player, and two U17 international players.

No formal a priori sample size calculation was performed. The study was exploratory and recruitment was based on the availability of elite players meeting the predefined eligibility criteria during the study period. Given the highly selected nature of this population and the limited accessibility of internationally experienced athletes, all eligible players who were available and agreed to participate were included.

Demographic, anthropometric, and sport-related characteristics were recorded for all participants, including age, height, body mass, sport experience, weekly training volume, mean daily training duration, rating of perceived exertion (RPE), and session rating of perceived exertion (sRPE) during the week of testing. Session-RPE was calculated by multiplying training duration by the corresponding RPE value and was expressed in arbitrary units (AU).

The inclusion criteria were as follows: (1) international competitive experience representing a national team; (2) regular participation in high-level national or international table tennis competitions; (3) systematic participation in table tennis training programmes; and (4) absence of injury or medical condition that could affect performance during the assessment. Players were excluded if they reported any acute musculoskeletal injury, neurological disorder, uncorrected visual condition, or other health-related limitation that could interfere with the execution of the test or with the quality of the eye-tracking recording. Participants performed the experimental assessment using their habitual optical correction when required.

All participants were free from injury at the time of testing. Written informed consent was obtained from adult participants and from the parents or legal guardians of participants under 18 years of age before participation. The study was conducted in accordance with the Declaration of Helsinki and approved by the Research Ethics Committee for Medicinal Products of Hospital Clínico San Carlos, Madrid, Spain (approval code: C.I. 23/415-E; date of approval: 28 June 2023).

### 2.3. Experimental Procedure

All assessments were performed individually in the table tennis hall of the CAR Madrid. Participants were evaluated in a single session and under homogeneous indoor conditions. Before testing, participants completed a standardised 10-min warm-up consisting of joint mobility exercises, low-intensity displacements, muscular activation, high-intensity displacements, and plyometric exercises. This was followed by 2 min of table-specific practice, reproducing their usual pre-competition warm-up routine.

After the warm-up, the wearable eye-tracking system was fitted to each participant and adjusted individually to ensure a stable position during the task. Before starting the experimental test, the eye-camera view was checked to confirm adequate visualisation of both eyes, and the interpupillary distance was adjusted when required. According to the manufacturer, the Pupil Labs Neon system does not require a conventional calibration procedure before or during recording. Participants then completed the sport-specific table tennis performance test described below.

Each participant performed a single experimental series. The protocol consisted of 56 balls delivered by a table tennis robot. The first six balls were used as familiarisation trials and were not included in the main analysis. These familiarisation trials were identified in the eye-tracking recordings as the initial practice phase. The remaining 50 forehand topspin strokes constituted the analysed test phase and were used for the extraction of ocular and technical performance variables. The analysed phase lasted approximately 3 min, with minor individual variation related to the execution of the protocol.

The same sequence, robot settings, ball-delivery frequency, target configuration, and scoring procedure were used for all participants to ensure standardisation of the experimental protocol.

To reduce the influence of potential modulators of ocular activity, participants were instructed to avoid caffeine intake for at least 2 h before testing. All recordings were obtained in the same indoor facility without natural light exposure and within the same time period, thereby reducing between-session variation in environmental illumination and circadian influences on pupil-related measures. Ambient illumination was not quantified in lux during data collection.

Figure 1 illustrates the experimental set-up used during the table tennis-specific performance test, including the player’s position, the wearable eye-tracking system, and the robot-based ball delivery configuration.

To facilitate interpretation of the experimental workflow, Figure 2 provides an overview of the complete procedure, from participant eligibility screening and standardised warm-up to simultaneous ocular and technical performance recording, data processing, and statistical analysis.

### 2.4. Eye-Tracking Assessment

Ocular variables were recorded using a wearable eye-tracking system (Pupil Labs Neon, Pupil Labs GmbH, Berlin, Germany). According to the manufacturer’s technical specifications, the system supports binocular and monocular eye tracking and provides 2D gaze data at 200 Hz, infrared eye video at 200 Hz, RGB scene video at 30 Hz, pupillometry data and eye state at 200 Hz, as well as eye position, eye orientation, pupil diameter, fixation data, and blink data. The device is designed as a calibration-free system and therefore does not require a conventional calibration procedure before or during recording.

In addition, the device includes inertial sensors, such as gyroscopes and accelerometers, which allow head-motion data to be recorded. Inertial sensor data were not included in the main analysis of the present study.

The eye tracker was fitted before the experimental test and adjusted individually for each participant. Before each recording, the eye-camera view was checked to confirm adequate visualisation of both eyes and stable positioning of the device. Recordings were managed in real-time using the Neon Companion app (version 2.9.31; Pupil Labs GmbH, Berlin, Germany) and processed post hoc using Pupil Cloud, a web-based platform provided by Pupil Labs GmbH.

During data extraction, the recordings and exported datasets were reviewed to verify adequate eye-camera visualisation and the availability of the ocular variables required for the 50 analysed forehand topspin strokes. Recordings were checked for incomplete acquisition, interruptions in eye visualisation, and incomplete variable export. A variable was considered suitable for analysis when the corresponding recording and exported data allowed its assessment across the analysed test phase.

Frame-level tracking-loss percentages were not retained prospectively in the dataset used for the present analysis. Data quality was therefore assessed by reviewing the recordings and verifying the availability and continuity of the exported ocular variables. Missing values were not imputed.

The ocular variables extracted for the present analysis were total blink count, blink frequency, mean blink duration, mean pupil diameter, maximum pupil diameter, and minimum pupil diameter. Blink frequency was expressed as blinks·min^−1^ and blink duration in milliseconds. Pupil-diameter metrics were obtained from the binocular 3D eye-state data exported from Pupil Cloud.

Individual blink-duration data were available for a subset of 12 participants and were therefore analysed descriptively only. No imputation was performed for unavailable blink-duration data.

### 2.5. Sport-Specific Technical Performance Test

Technical performance was assessed using a modified version of the table tennis stroke–performance test described by Le Mansec et al. [15]. The test was performed on a Butterfly Octet 25+ table (Butterfly, Tokyo, Japan) using a NARAQ RoboPing Halo Pro table tennis robot (Future Mind, Shanghai, China). The robot delivered 56 backspin balls at a velocity of 35 km·h^−1^ and a frequency of one ball every 3 s. The first six balls were used as familiarisation trials and were excluded from the analysis. Therefore, a total of 50 valid forehand topspin strokes were analysed for each participant.

The spatial arrangement of the players, the target zones, and the general configuration of the test followed the protocol described by Le Mansec et al. [15], with the adaptations required for the present experimental setting. Participants were instructed to perform forehand topspin strokes and to direct the ball towards the established target area.

Ball velocity was recorded using a Stalker Pro IIs radar gun (Applied Concepts Inc., Richardson, TX, USA), configured with a sampling frequency of 50 Hz and an accuracy of ±0.041 m·s^−1^. The radar was positioned 1 m behind the table, aligned with the longitudinal axis of the robot, and at a height of 113 cm. Mean ball velocity was calculated from the 50 analysed strokes.

Stroke accuracy was assessed using the scoring system proposed by Le Mansec et al. [15]. Each stroke received two points when the ball landed within the target area, one point when the ball landed on the table outside the target area, and zero points when the ball did not land on the playing surface. The total accuracy score was calculated as the sum of the 50 analysed strokes, with higher scores indicating better stroke accuracy.

These procedures were applied consistently across participants, and the use of predefined robot settings, target zones, scoring criteria, and radar positioning was intended to facilitate replication of the test under comparable experimental conditions.

### 2.6. Statistical Analysis

Statistical analyses were performed using Python 3.13. Descriptive statistics were calculated using pandas version 3.0.1, correlation analyses were conducted using SciPy version 1.17.1, and figures were generated using Matplotlib version 3.10.8. Continuous variables are presented as the mean ± standard deviation when normally distributed, and as median and interquartile range when normality assumptions were not met. The normality of the data distribution was assessed using the Shapiro–Wilk test and visual inspection of the histograms and Q–Q plots.

Analyses were conducted using the maximum number of valid observations available for each variable. Participants with unavailable data for a specific ocular variable were excluded only from analyses involving that variable. Missing eye-tracking values were not imputed.

Given the exploratory nature of the study and the limited sample size, correlation analyses were interpreted cautiously, with particular attention to the magnitude and direction of the observed associations. Statistical significance was set at *p* < 0.05.

To account for the number of correlation tests performed, the Benjamini–Hochberg procedure was applied across the 10 correlation analyses as a sensitivity analysis to control the false discovery rate (FDR). Both unadjusted and FDR-adjusted *p*-values are reported.

To quantify the uncertainty surrounding the correlation estimates, 95% confidence intervals were calculated using bias-corrected and accelerated (BCa) bootstrap resampling with 10,000 iterations. Confidence intervals were calculated separately for each Pearson or Spearman correlation using the available paired observations for the corresponding analysis.

The main associations of interest were those between the blink-related variables and performance outcomes, and between the pupil-related variables and performance outcomes. Technical performance outcomes were stroke accuracy and mean ball velocity.

Given the exploratory design and the limited availability of internationally experienced elite players, no formal a priori sample size calculation was performed. To characterise the statistical sensitivity of the available sample, a sensitivity analysis was conducted for a two-sided correlation test with α = 0.05 and 80% power. With the sample sizes available for the principal correlation analyses (*n* = 15–16), the minimum detectable correlation was approximately |r| = 0.64 for *n* = 15 and |r| = 0.63 for *n* = 16. Accordingly, the study was primarily sensitive to relatively large associations, and smaller or moderate relationships may not have been detected.

## 3. Results

Technical performance data were available for all 17 participants. Blink count and blink-frequency data from the analysed test phase were available for 16 participants (94.1%), whereas individual blink-duration data were available for 12 participants (70.6%). Pupil-diameter data were available for 15 participants (88.2%). One participant had no usable post-test eye-tracking dataset for the ocular analyses, and pupil-diameter data were unavailable for one additional participant in the exported 3D eye-state dataset. Accordingly, analyses were performed using the maximum number of valid observations available for each variable, with no imputation of missing data.

### 3.1. Participant Characteristics

The participants had a mean age of 20.34 ± 3.86 years, height of 177.91 ± 3.79 cm, body mass of 69.97 ± 8.12 kg, body mass index (BMI) of 23.13 ± 2.42 kg·m^−2^, sport experience of 11.36 ± 4.37 years, and weekly training volume of 21.91 ± 3.83 h·week^−1^. Training load during the week of testing was additionally characterised by mean daily training duration, rating of perceived exertion (RPE), and session-RPE (sRPE), as shown in Table 1.

### 3.2. Descriptive Analysis of Ocular and Technical Performance Variables

During the analysed test phase, participants achieved a mean stroke accuracy score of 80.47 ± 3.04 points, with scores ranging from 75 to 86 points. Mean ball velocity was 48.83 ± 3.79 km·h^−1^, ranging from 42.59 to 55.33 km·h^−1^.

Regarding blink-related variables, the mean total blink count was 50.56 ± 36.53 blinks during the analysed test phase, whereas the mean blink frequency was 16.79 ± 11.57 blinks·min^−1^. Mean pupil diameter was 4.71 ± 0.45 mm. The mean maximum pupil diameter was 5.77 ± 0.57 mm, and the mean minimum pupil diameter was 1.60 ± 0.14 mm. A complete summary of the ocular and technical performance variables is provided in Table 2.

Individual blink-duration data were available for 12 participants. Mean blink duration was 261.10 ± 28.17 ms, with values ranging from 216.51 to 312.13 ms (Table 3).

### 3.3. Associations Between Ocular Variables and Technical Performance

Correlation analyses between ocular variables and technical performance outcomes are presented in Table 4. Blink frequency showed the strongest positive association with stroke accuracy score (Spearman’s ρ = 0.613, 95% CI [0.060, 0.881], *p* = 0.012); however, this association did not remain statistically significant after FDR correction (FDR-adjusted *p* = 0.115). Total blink count showed a positive association in the same direction (ρ = 0.496, 95% CI [−0.012, 0.824], *p* = 0.051; FDR-adjusted *p* = 0.254). Neither blink-related variable was significantly associated with ball velocity.

No clear associations were observed between the pupil-related variables and either stroke accuracy or ball velocity. Mean pupil diameter showed little association with stroke accuracy (r = −0.130, 95% CI [−0.524, 0.459], *p* = 0.645) or ball velocity (r = 0.060, 95% CI [−0.606, 0.638], *p* = 0.832). Similarly, maximum and minimum pupil diameter showed no statistically significant associations with either technical performance outcome.

The relationship between blink-related variables and stroke accuracy score is shown in Figure 3. Both the total blink count and blink frequency showed positive associations with stroke accuracy. The association with blink frequency reached nominal statistical significance before multiple-comparison correction but did not remain significant after FDR adjustment. This pattern should therefore be interpreted as exploratory rather than confirmatory.

The relationship between mean pupil diameter and stroke accuracy score is shown in Figure 4. In contrast to blink frequency, mean pupil diameter was not significantly associated with stroke accuracy (Pearson’s r = −0.130, *p* = 0.645; *n* = 15). The scatterplot did not show a clear linear trend, consistent with the lack of a clear association between the mean pupil diameter and accuracy performance in the present sample.

Supplementary Figure S1 shows scatterplots of the mean, maximum, and minimum pupil diameter in relation to stroke accuracy and ball velocity, with no clear visual trends apparent across these associations.

Overall, blink frequency showed the strongest association with stroke accuracy, whereas no comparable pattern was observed for pupil-diameter measures or ball velocity. No relevant associations were observed between the ocular variables and ball velocity.

## 4. Discussion

The present study analysed blink behaviour and pupil diameter recorded with wearable eye-tracking technology during a sport-specific table tennis test in elite male players. Blink frequency showed the strongest observed association with stroke accuracy, although this association did not remain statistically significant after correction for multiple comparisons. Total blink count showed a weaker association in the same direction. No clear associations were observed between the global pupil-diameter measures and either stroke accuracy or ball velocity. Overall, these findings should be interpreted cautiously given the exploratory design, limited sample size, and multiple correlations examined.

The positive association observed between blink frequency and stroke accuracy is of interest in a sport that requires the continuous processing of rapidly changing visual information and precise visuomotor coordination. Spontaneous blinking has a fundamental physiological role in maintaining ocular-surface integrity and visual function, but it is also influenced by attention, cognitive state, and task demands [10,11,18,19,20,21]. Experimental studies have shown that blink occurrence can vary according to the temporal structure of a task, with blinks tending to be suppressed when relevant information is expected and occurring more frequently during periods of reduced information demand or after response execution [19,20,21].

These findings provide a plausible context for the present association, but they do not establish its underlying mechanism. We analysed overall blink frequency rather than the timing of individual blinks relative to ball delivery, ball flight, or racket–ball contact. It is therefore not possible to determine whether the more accurate players distributed their blinks differently across the stroke sequence or whether the association reflects other physiological, attentional, or individual factors. Time-resolved analyses will be required to determine whether blink suppression or displacement occurs around visually critical phases of table tennis performance.

Total blink count showed a positive association with stroke accuracy in the same direction as blink frequency, although this association did not reach statistical significance. Blink frequency may nevertheless be the more informative descriptive measure because it accounts for recording duration, whereas absolute blink count is directly influenced by the length of the analysed interval. Given the small sample and the exploratory nature of the analyses, these associations should be interpreted primarily in terms of their magnitude and uncertainty rather than as evidence of a reproducible performance marker.

In contrast, blink-related variables were not associated with ball velocity. This suggests that the ocular variables analysed in the present study were more closely related to accuracy than to the speed component of performance. Stroke accuracy and ball velocity may depend on partially different determinants. Accuracy is likely to require fine visuomotor control, spatial precision, and appropriate use of visual information, whereas ball velocity may be more strongly influenced by biomechanical, coordinative, and neuromuscular factors. Previous work using table tennis-specific performance tests has shown that accuracy and speed can be affected differently by fatigue and task demands [14,15]. Therefore, the absence of an association between blink behaviour and ball velocity is not unexpected and may indicate that blink-related metrics are more informative for perceptual accuracy than for power-related technical output.

No associations were observed between the global pupil-diameter measures and either stroke accuracy or ball velocity. This result is limited, however, to the mean, maximum, and minimum pupil values examined here and does not exclude a relationship between transient pupillary responses and table tennis performance. Pupil diameter is sensitive to cognitive load, mental effort, and arousal, but also to non-cognitive influences such as illumination, accommodation, fatigue, emotional state, individual variability, and recording conditions [12,13,17,22,23].

Averaging pupil diameter across the complete test phase may obscure short-lived responses associated with specific moments of the task. Changes occurring around ball delivery, anticipation, stroke preparation, racket–ball contact, or recovery may not be reflected adequately by overall mean, maximum, or minimum values. Event-related pupillometry and time-locked analyses of pupil dynamics would therefore be more appropriate for determining whether transient pupillary responses are related to performance during table tennis. Accordingly, the present results only apply to the global pupil metrics examined here.

Wearable eye tracking is increasingly being used to study visuomotor behaviour under more representative sport conditions. In table tennis, previous work has mainly focused on gaze behaviour, eye and head movements, and visuomotor strategies during naturalistic or sport-specific tasks [5,6,9]. Our study adds a different perspective by examining blink behaviour and pupil diameter, which have received comparatively less attention in this sport. Recording these variables during a standardised stroke–performance test allowed them to be assessed without removing the temporal and technical constraints of the task. This may be useful for narrowing the gap between laboratory-based visual assessment and applied performance evaluation.

Recent reviews have emphasised the need to move sport eye-tracking research towards more representative tasks, improved data-processing approaches, and field-based designs that preserve the constraints of real performance environments [24,25].

Wearable eye tracking also makes it possible to record blink behaviour repeatedly during sport-specific drills with little disruption to the task. The present results do not support using blink frequency as a stand-alone marker of performance, alertness, or fatigue, and they certainly do not imply that athletes should try to modify their blink rate. A more useful direction may be repeated within-player monitoring under standardised conditions, comparing changes in blink behaviour with technical accuracy, perceived fatigue, training load, and other eye-tracking measures. Whether this provides meaningful information for coaches or sport scientists will depend on demonstrating adequate reliability, sensitivity to change, and responsiveness to fatigue or training load.

The study has several limitations that should be considered when interpreting these findings. The sample size was small, reflecting the difficulty of recruiting elite table tennis players with international competitive experience, and this limits the precision and generalisability of the estimated associations. The sensitivity analysis indicated that the available sample was primarily capable of detecting relatively large correlations; therefore, smaller or moderate associations cannot be excluded. The high competitive level of the sample may also have restricted the range of technical performance scores, particularly stroke accuracy. In addition, blink count showed substantial between-participant variability, which may reflect genuine differences in spontaneous blink behaviour, ocular-surface characteristics, attentional state, fatigue, or task strategy; however, measurement-related variability during dynamic wearable eye tracking cannot be excluded. Test–retest reliability was not assessed, so the relative contribution of biological and measurement variability cannot be determined from the present data. The inclusion of only male players limits generalisability to female athletes. The study was designed to examine associations within an elite population rather than differences between expertise levels, and therefore did not include a recreational or non-elite comparison group. Consequently, the present findings cannot determine whether blink behaviour or pupil-diameter characteristics differ according to competitive level or sporting expertise. Finally, the cross-sectional design prevents causal interpretation of the observed relationships.

Some methodological limitations are also relevant to the ocular measurements. Blink count and blink-frequency data were available for 16 participants, blink-duration data for 12 participants, and pupil-diameter data for 15 participants, resulting in variable sample sizes across analyses. Ambient illumination was kept as consistent as possible by conducting all assessments in the same indoor facility, without natural light exposure and within the same time period, but illumination was not quantified in lux. Dietary intake, nutritional supplement use, and screen or social-media exposure before testing were not formally monitored. In addition, the study analysed global blink and pupil metrics across the complete test phase rather than their temporal relationship with specific events within the stroke sequence.

Finally, multiple correlations were performed within an exploratory framework, increasing the risk of type I error despite the FDR sensitivity analysis.

This cautious interpretation is especially important in sport and exercise science, where small samples and selective reporting can contribute to unstable effect-size estimates and replication concerns [26].

Larger and more diverse samples will be needed to determine whether these exploratory associations are reproducible, particularly in female players and across different competitive levels. Repeated-measures and longitudinal designs could also establish the test–retest reliability and within-athlete stability of blink-related metrics and clarify whether they change with fatigue, training load, or perceptual-cognitive interventions. Beyond this, analysing blink timing and event-related pupil responses in relation to specific phases of the stroke sequence—such as ball delivery, approach, racket–ball contact, and recovery—would provide more direct information than global ocular measures. Combining time-resolved eye tracking with biomechanical, physiological, and performance data may ultimately offer a more complete account of visual behaviour during high-level table tennis performance.

In this context, approaches based on dynamic areas of interest and time-resolved analysis of head-mounted eye-tracking data may be especially useful for examining how ocular behaviour changes across the rapidly evolving visual events of table tennis [27].

## 5. Conclusions

The present study provides preliminary evidence that blink behaviour may contain complementary information about technical performance during a standardised table tennis task in elite male players. The proposed hypothesis was only partially supported: blink-related measures showed the clearest relationship with stroke accuracy, whereas the global pupil-diameter measures examined did not show a comparable relationship with the selected performance outcomes.

These findings should be regarded as exploratory and hypothesis-generating rather than confirmatory. They do not establish a causal or mechanistic role for blinking in performance, nor do they currently support the use of blink frequency as an independent training or fatigue marker. Time-resolved analysis, reliability assessment, and replication in larger and more diverse samples are needed to determine whether blink-related metrics can provide useful information for athlete monitoring or performance assessment.

## Figures and Tables

**Figure 1:**
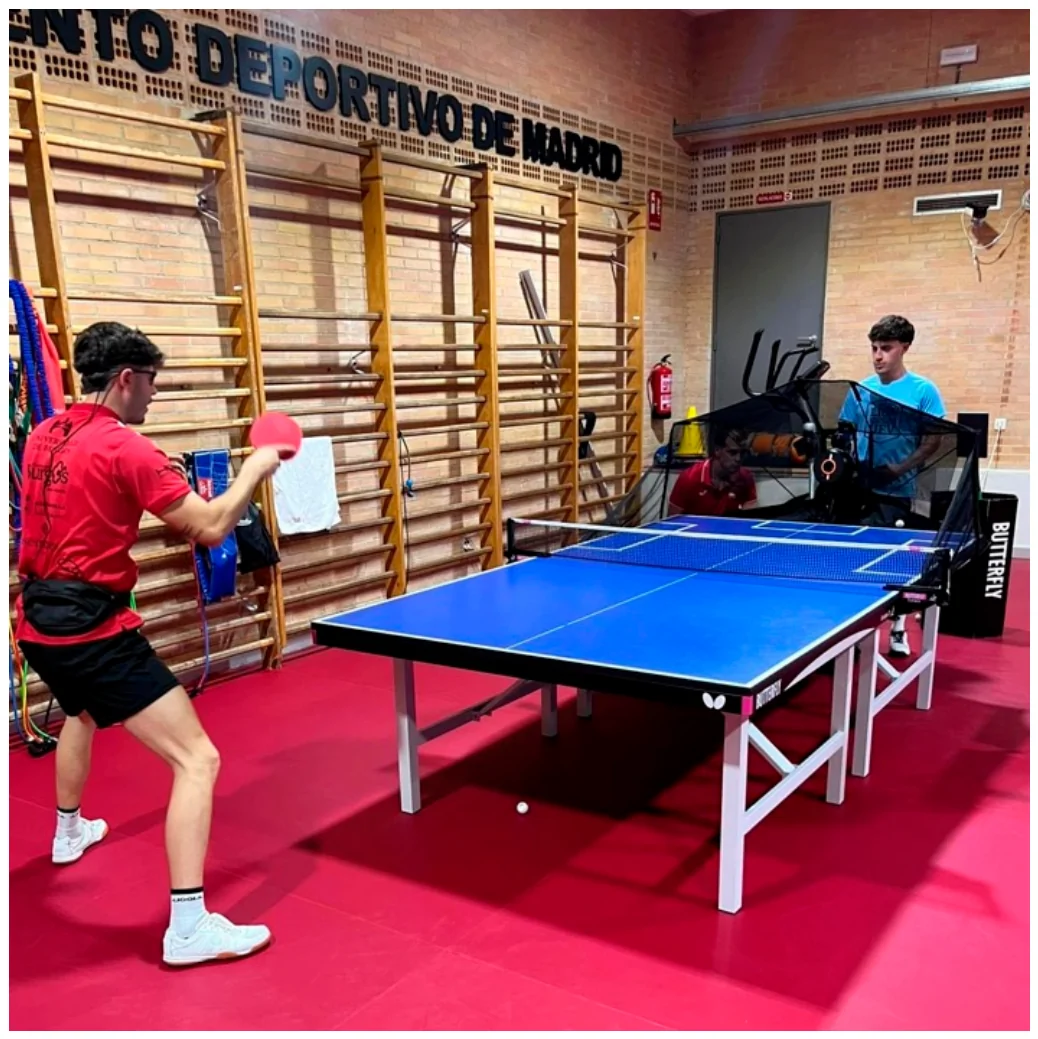
Experimental set-up of the table tennis-specific performance test.

**Figure 2:**
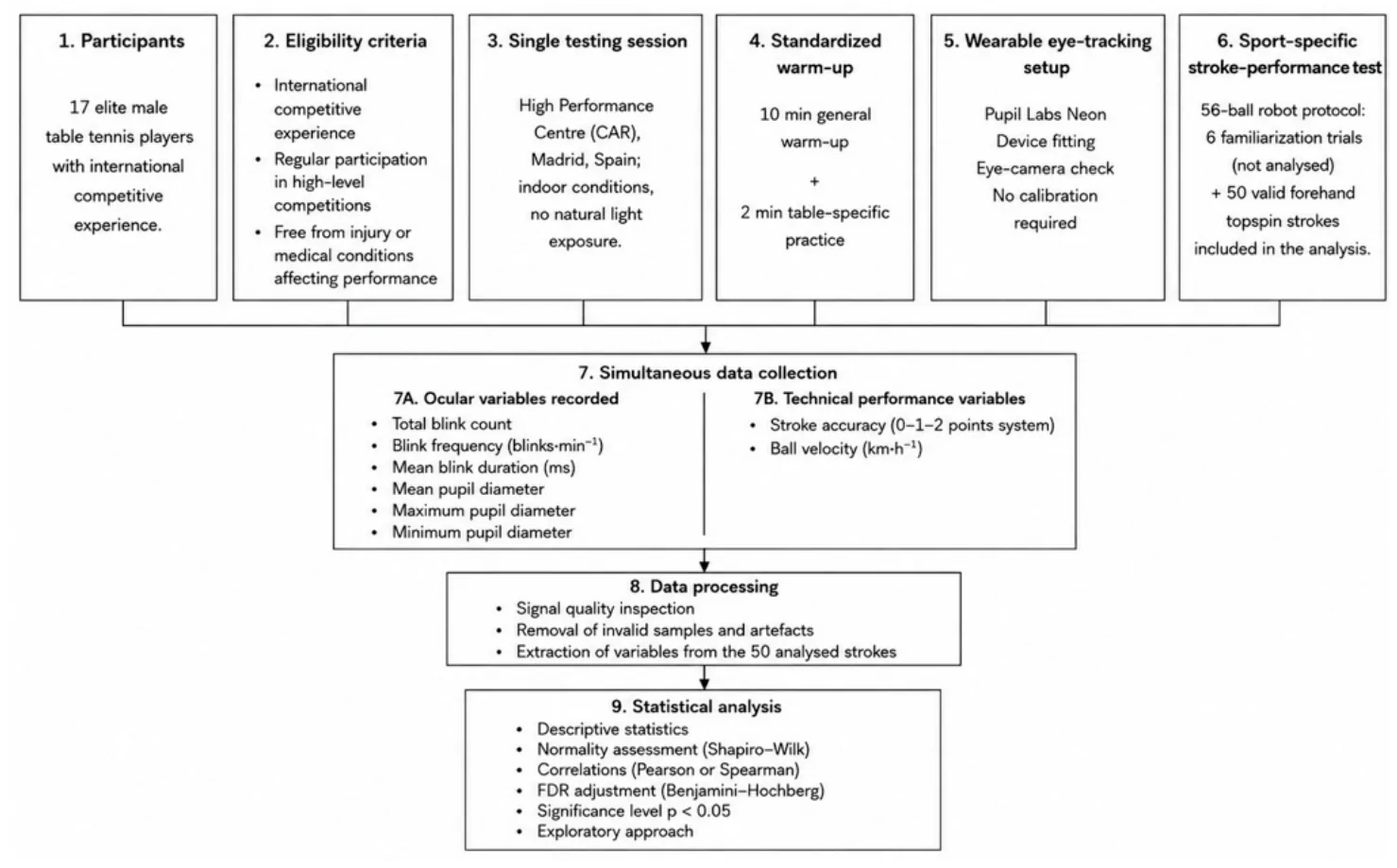
Schematic overview of the experimental procedure.

**Figure 3:**
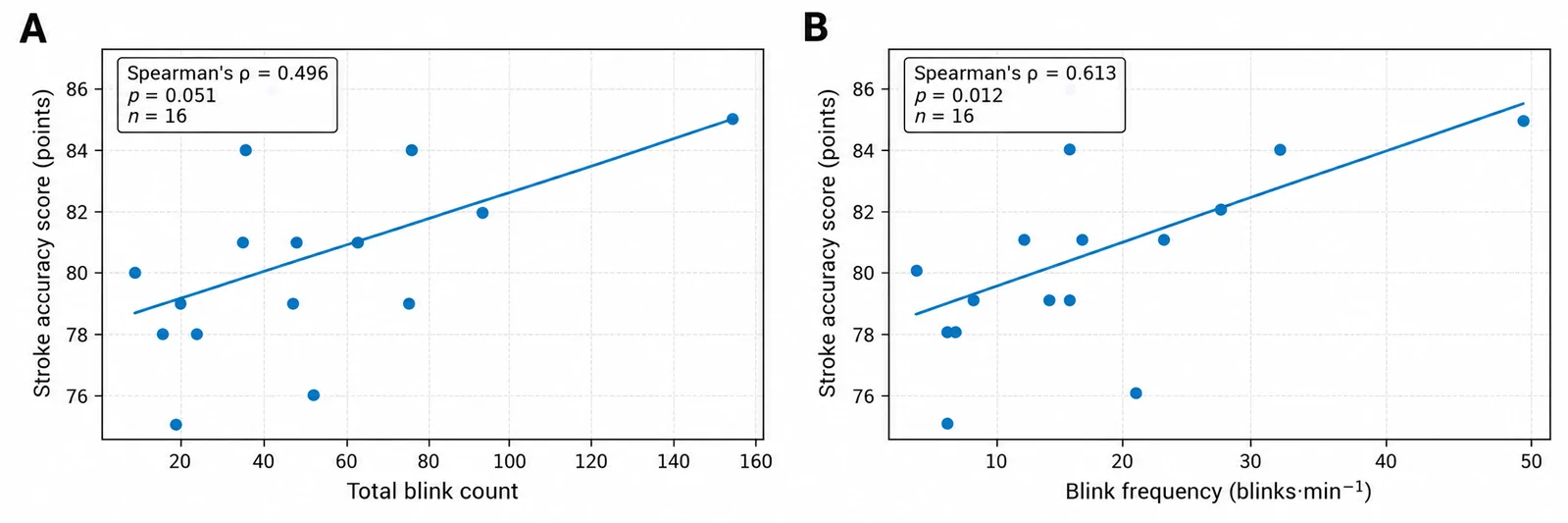
Associations between blink-related variables and stroke accuracy score during the analysed test phase. (**A**) Total blink count. (**B**) Blink frequency.

**Figure 4:**
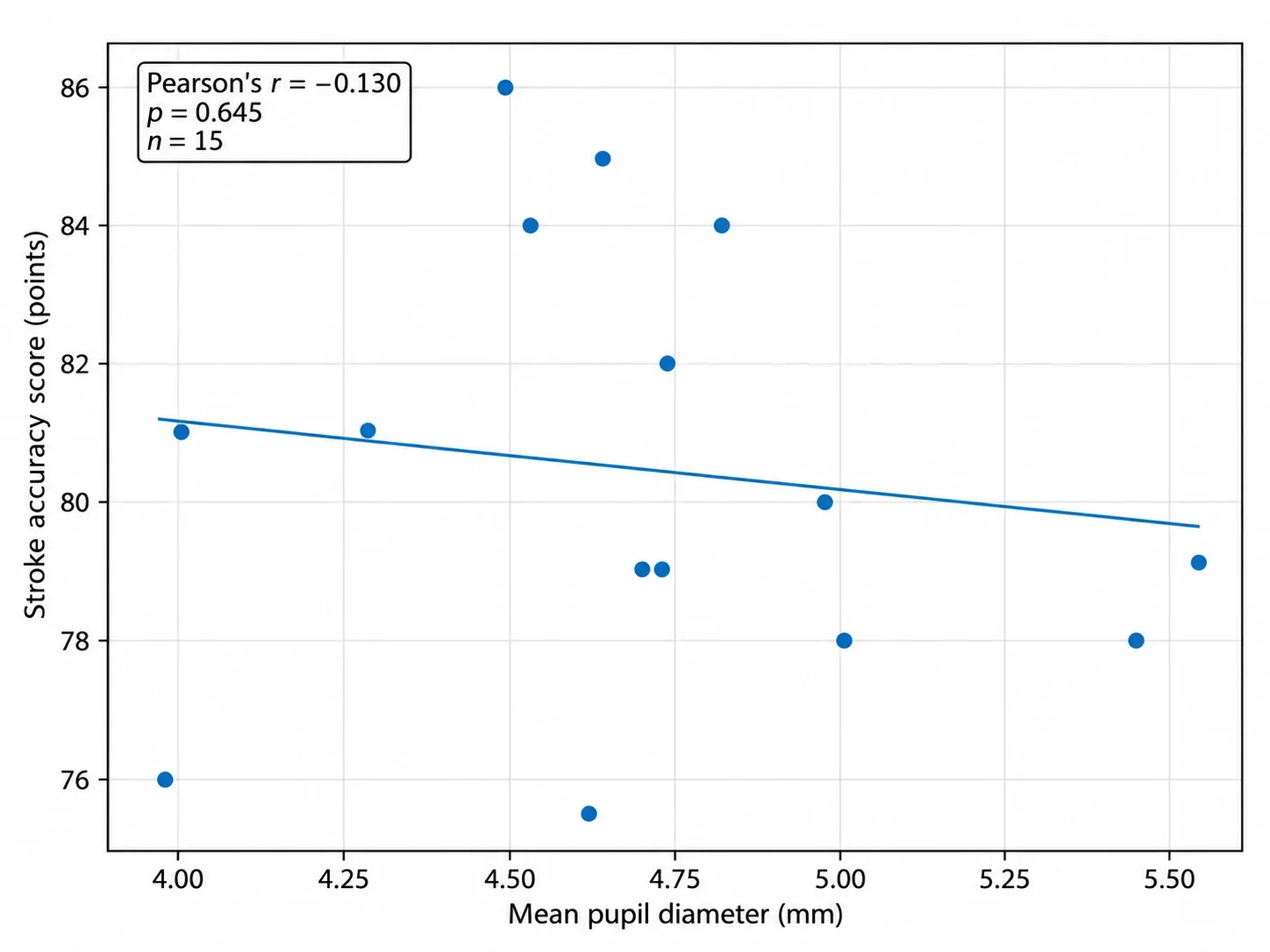
Relationship between the mean pupil diameter and stroke accuracy score during the analysed test phase.

**Table 1:** Descriptive characteristics of the sample.

Variable	*n*	Mean ± SD
Age, years	17	20.34 ± 3.86
Height, cm	17	177.91 ± 3.79
Body mass, kg	17	69.97 ± 8.12
Body mass index, kg·m^−2^	17	23.13 ± 2.42
Sport experience, years	17	11.36 ± 4.37
Weekly training volume, h·week^−1^	17	21.91 ± 3.83
Mean daily training duration, min·day^−1^	17	139.60 ± 29.44
Rating of perceived exertion (RPE)	17	6.29 ± 1.04
Session-RPE (sRPE), AU	17	880.53 ± 262.59

Note: SD = standard deviation; RPE = rating of perceived exertion; sRPE = session rating of perceived exertion; AU = arbitrary units.

**Table 2:** Descriptive statistics of the ocular and technical performance variables during the analysed test phase.

Variable	*n*	Mean ± SD	Median	IQR	Minimum	Maximum
Total blink count	16	50.56 ± 36.53	45.00	43.00	9.00	154.00
Blink frequency, blinks·min^−1^	16	16.79 ± 11.57	15.07	12.82	3.67	49.27
Mean pupil diameter, mm	15	4.71 ± 0.45	4.70	0.39	3.98	5.62
Maximum pupil diameter, mm	15	5.77 ± 0.57	5.57	0.30	5.24	7.41
Minimum pupil diameter, mm	15	1.60 ± 0.14	1.63	0.23	1.36	1.80
Stroke accuracy score, points	17	80.47 ± 3.04	80.00	3.00	75.00	86.00
Ball velocity, km·h^−1^	17	48.83 ± 3.79	48.35	4.57	42.59	55.33

Note: Sample size varies according to data availability: *n* = 17 for technical performance variables, *n* = 16 for total blink count and blink frequency, and *n* = 15 for pupil-diameter variables. Blink-duration data (*n* = 12) are reported separately in Table 3. SD, standard deviation; IQR, interquartile range.

**Table 3:** Descriptive characteristics of blink duration during the analysed table tennis performance test.

Variable	*n*	Mean ± SD	Median (IQR)	Range
Mean blink duration (ms)	12	261.10 ± 28.17	258.30 (30.69)	216.51–312.13

SD, standard deviation; IQR, interquartile range.

**Table 4:** Correlations between the ocular variables and technical performance outcomes.

Ocular Variable	Performance Outcome	*n*	Test	Correlation Coefficient	95% CI	*p*-Value	FDR-Adjusted *p*-Value
Total blink count	Stroke accuracy score	16	Spearman	*ρ* = 0.496	(−0.012, 0.824)	0.051	0.254
Total blink count	Ball velocity	16	Spearman	ρ = −0.130	(−0.535, 0.340)	0.633	0.807
Blink frequency	Stroke accuracy score	16	Spearman	*ρ* = 0.613	(−0.060, 0.881)	0.012	0.115
Blink frequency	Ball velocity	16	Spearman	ρ = −0.129	(−0.578, 0.368)	0.633	0.807
Mean pupil diameter	Stroke accuracy score	15	Pearson	*r* = −0.130	(−0.524, 0.459)	0.645	0.807
Mean pupil diameter	Ball velocity	15	Pearson	*r* = 0.060	(−0.606, 0.638)	0.832	0.869
Maximum pupil diameter	Stroke accuracy score	15	Spearman	*ρ* = −0.253	(−0.724, 0.438)	0.362	0.807
Maximum pupil diameter	Ball velocity	15	Spearman	*ρ* = −0.046	(−0.629, 0.489)	0.869	0.869
Minimum pupil diameter	Stroke accuracy score	15	Pearson	*r* = −0.138	(−0.676, 0.510)	0.623	0.807
Minimum pupil diameter	Ball velocity	15	Pearson	*r* = 0.327	(−0.286, 0.699)	0.234	0.781

Note: 95% confidence intervals were calculated using bias-corrected and accelerated (BCa) bootstrap resampling with 10,000 iterations. Sample size varies according to the availability of each ocular variable. Missing data were not imputed. FDR-adjusted *p*-values were calculated using the Benjamini–Hochberg procedure across the 10 correlation tests. Bold indicates statistically significant unadjusted *p*-values (*p* < 0.05). *r*, Pearson correlation coefficient; *ρ*, Spearman rank correlation coefficient.

## Data Availability

The data supporting the findings of this study are available from the corresponding author upon reasonable request. The data are not publicly available due to privacy and ethical restrictions related to participant-level information.

## Supplementary Materials

The following supporting information can be downloaded at: https://www.mdpi.com/article/10.3390/sports14100420/s1, Figure S1. Associations between pupil diameter variables and technical performance outcomes.

## Abbreviations

The following abbreviations are used in this manuscript:

CAR	High Performance Centre
CEIm	Research Ethics Committee for Medicinal Products
C.I.	Internal code
IQR	Interquartile range
RGB	Red, green, and blue
SD	Standard deviation
