# Supplementary material for: Blink Behaviour, Pupil Diameter, and Technical Performance in Elite Male Table Tennis Players: A Wearable Eye-Tracking Study
Source: Sports (Basel). 2026 Oct 1;14(10):420. doi: 10.3390/sports14100420 (PMC13709445; doi:10.3390/sports14100420)

Figure S1. Associations between pupil diameter variables and technical performance outcomes.

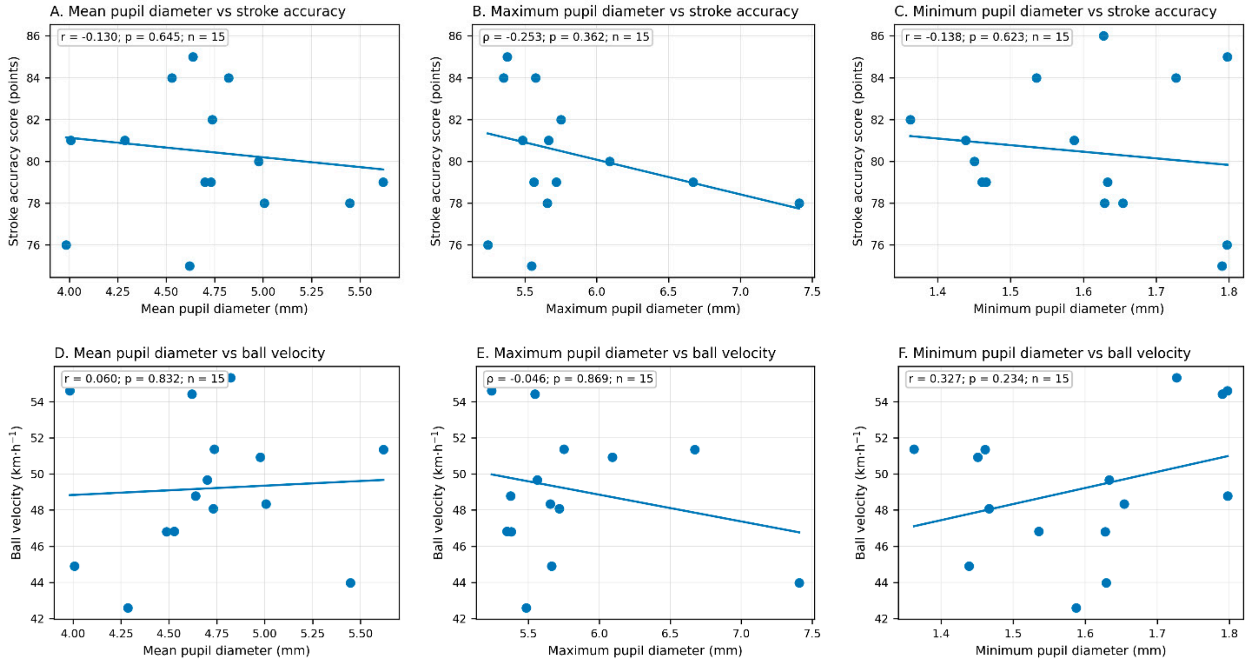

Supplement: Supplementary file 1 [file sports-14-00420-s001.zip › sports-4438875-supplementary.pdf]
